# Altitude, habitat type and herbivore damage interact in their effects on plant population dynamics

**DOI:** 10.1371/journal.pone.0209149

**Published:** 2018-12-17

**Authors:** Tomáš Dostálek, Maan Bahadur Rokaya, Zuzana Münzbergová

**Affiliations:** 1 Institute of Botany, the Czech Academy of Sciences, Průhonice, Czech Republic; 2 Department of Botany, Faculty of Science, Charles University, Prague, Czech Republic; 3 Department of Biodiversity Research, Global Change Research Centre, the Czech Academy of Sciences, Brno, Czech Republic; University of Michigan, UNITED STATES

## Abstract

Insects represent one of the most abundant groups of herbivores, and many of them have significant impacts on the dynamics of plant populations. As insects are very sensitive to changes in climatic conditions, we hypothesize that their effects on plant population dynamics will depend on climatic conditions. Knowledge of the variation in herbivore effects on plant population dynamics is, however, still rather sparse. We studied population dynamics and herbivore damage at the individual plant level of *Salvia nubicola* along a wide altitudinal gradient representing a range of climatic conditions. Using integral projection models, we estimated the effect of changes in herbivore pressure on plant populations in different climates and habitat types. Since we recorded large differences in the extent of herbivore damage along the altitudinal gradient, we expected that the performance of plants from different altitudes would be affected to different degrees by herbivores. Indeed, we found that populations from low altitudes were better able to withstand increased herbivore damage, while populations from high altitudes were suppressed by herbivores. However, the pattern described above was evident only in populations from open habitats. In forest habitats, the differences in population dynamics between low and high altitudes were largely diminished. The effects of herbivores on plants from different altitudes were thus largely habitat specific. Our results indicate potential problems for plant populations from high altitudes in open habitats because of increased herbivore damage. However, forest habitats may provide refuges for the plants at these high altitudes.

## Introduction

It has been estimated that herbivores consume approximately 10–20% of annual net primary production in terrestrial ecosystems [1]. As insect herbivores represent one of the most abundant groups of herbivores, they are thought to affect plant populations in a significant way [2,3]. Plants thus have developed a range of strategies that help them survive and maximize their fitness under herbivore pressure [4–8]. As insect herbivores are very sensitive to changes in climatic conditions [9], we hypothesize that the effects of herbivores on plant population dynamics will also differ under various climatic conditions [9,10]. Climate thus affects plant populations directly (e.g., phenology, growth and mortality) but also indirectly by shifting the outcomes of plant-herbivore interactions [10–12]. Therefore, we need to understand the variation in plant-herbivore interactions under different climatic conditions to be able to predict plant performance under varying climates [13].

The impacts of climate on plant populations are commonly studied along altitudinal gradients [14]. Most commonly, these studies explore how climate affects specific life history traits, such as phenology, ontogeny or reproduction [15–21]. However, changes in single life history traits may not be informative for the total population dynamics of the species (e.g., [22–25]). To identify the effects of climate on long-term species performance, we need to understand how different climatic conditions affect the total life cycle of the species [26].

Since the effect of herbivore damage on plant performance is also changing along climatic gradients [27], the effects of climate on plant population dynamics should be studied in combination with the effects of herbivore damage. To do this, we need to assess differences in plant population dynamics in different climatic conditions [26,28,29] in combination with the effects of herbivore damage on plant population dynamics [30–34]. While many studies have explored either plant population dynamics along altitudinal gradients or changes in plant-herbivore interactions, there have been very few studies combining both of these effects (but see [35]). Moreover, plant populations often exist in spatially heterogeneous environments, which can affect plant growth not only directly through resource availability [36] but also indirectly by altering the behaviour or success of insect herbivores [37]. Differences between habitats should thus be considered in studies on plant-herbivore interactions along altitudinal gradients.

In our study, we explored the effect of changes in plant-insect interactions along a gradient of climatic conditions on plant population dynamics. We collected data on the complete life cycle of *Salvia nubicola* in Nepal along a wide altitudinal range from 2100 to 3600 m a.s.l. Moreover, we recorded leaf herbivore damage by insects for each plant. Since habitat openness was also found to be a factor strongly affecting herbivore damage in this species [38] and varied independently of elevation, data were collected in both open and forest habitats.

We asked the following questions: 1) What is the effect of leaf herbivore damage on plant vital rates along a gradient of climatic conditions?, 2) How are these effects translated to differences in population growth rates?, and 3) Do the changes in the effects of herbivory on population dynamics along a climatic gradient depend on local habitat conditions? Since plants in low altitudes experience higher herbivore pressure and thus have more defences [8,38], we predicted that plants from lower altitudes are better adapted to herbivory than plants from higher altitudes. As a result, plants from a lower altitude will be more resistant to herbivore damage, and their population growth will thus be less affected by herbivory. The effects of herbivory on population growth at low altitudes will be even weaker in populations from open habitats than forest habitats since populations in open habitats were found to experience higher herbivore pressure in our previous study [38].

## Methods

### Study species

*Salvia nubicola* is a perennial iterocarpic herb that grows up to 60–150 cm and flowers from August to September. The distribution range of *S*. *nubicola* includes western and central Nepal, Afghanistan, Pakistan, northern India, Bhutan, and Tibet [39]. It grows in altitudes from 1800 to 3600 m a.s.l., often in open humus rich sites. The plants are usually not eaten by large herbivores but are severely damaged by insects [8]. Plant damage by insect herbivores varies along an altitudinal gradient [8,38]. In our previous study, we demonstrated that plants from lower altitudes suffer from higher herbivore pressure and are better defended against herbivores than plants from higher altitudes [8]. These factors make *S*. *nubicola* a perfect model species. Since *S*. *nubicola* is neither endangered nor protected and we only recorded observational data with no effect on the plant populations, no specific permissions were required.

### Studied populations

The study sites were selected in a valley in Manang, Annapurna Conservation Area in central Nepal where *S*. *nubicola* occurs frequently. The study sites ranged along an altitudinal gradient with strong differences in temperatures. There is a difference of 3°C in July and 9°C in January in mean month air temperature per 1000 m of altitude in this region [8]. Soil moisture decreases with altitude from east to west in the Manang Valley, and the south-facing slopes are significantly drier than those facing north [40]. The vegetation is dominated by *Pinus wallichiana*, which is abundant on the north aspect from the lower belt up to 3500 m a.s.l. *P*. *wallichiana* is replaced by *Abies spectabilis* and *Betula utilis* at higher altitudes. The habitats on the north facing slopes are thus highly shaded. *Juniperus indica* and *Rosa sericea* are the dominants on the dry south-facing slopes [41] with grazed grasslands, creating relatively open habitats.

**Fig 1:**
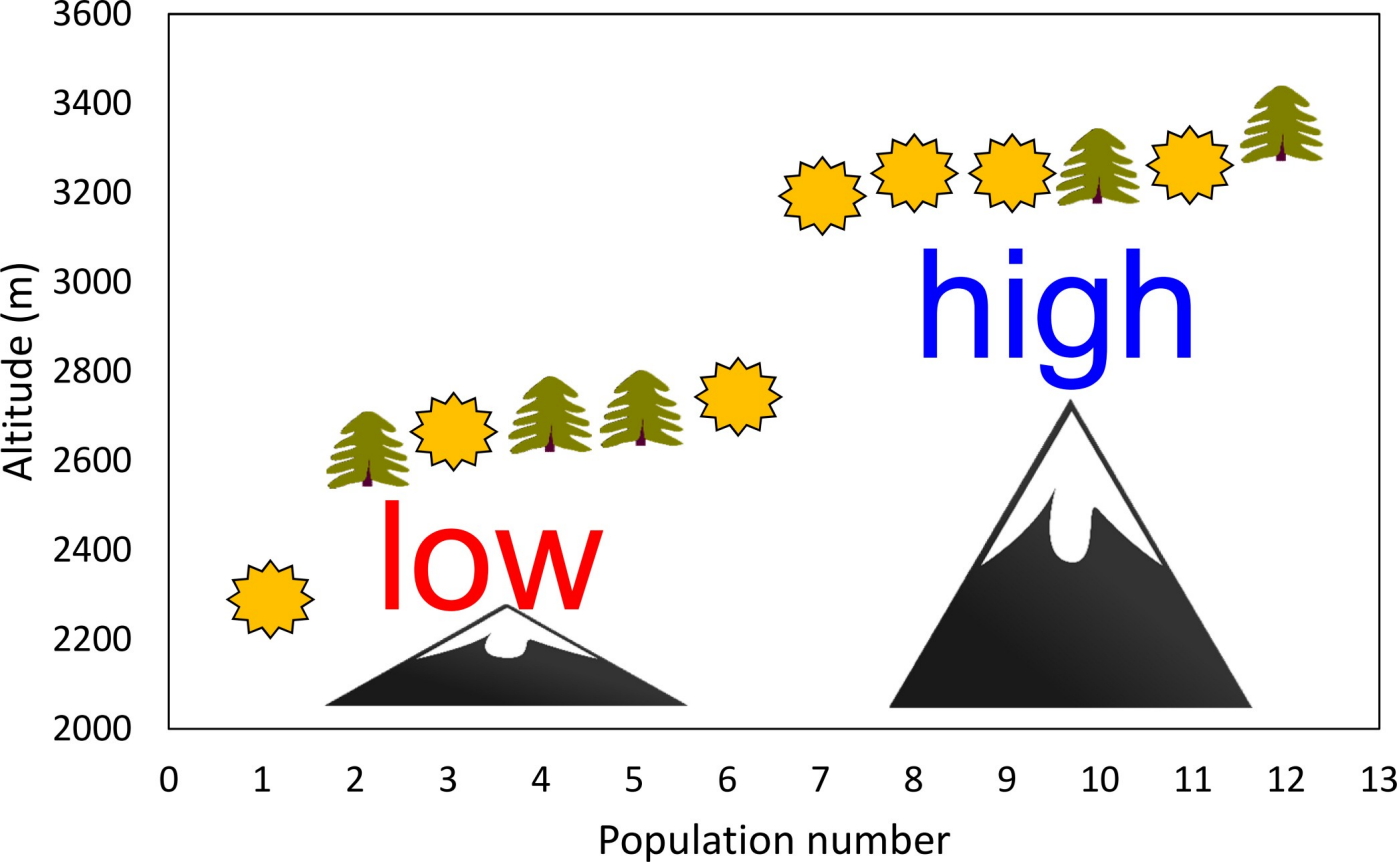
Schema of the experimental design. Distribution of the populations of *Salvia nubicola* along the altitudinal gradient used in the study. Populations in open habitats are indicated by “sun” symbols and in forest habitats by “tree” symbols. The population numbers correspond with codes in Table 1.

Altitude and habitat openness were found to be the factors that mostly affect herbivore damage in the study species [38]. The study populations were thus evenly distributed between those located at higher and lower altitudes (Fig 1 and Table 1). Within both lower and higher altitudes, we selected localities in open places and in forests (hereafter referred to as “open” and “forest”). The selected populations were at least 500 m apart and consisted of at least 100 individuals (except for one population with 70 individuals). Hereafter, combinations of altitude (high vs. low) and habitat openness (open vs. forest) are referred to as locality types.

**Table 1:** List of the 12 studied *Salvia nubicola* populations in the Annapurna Conservation Area, Nepal.

Pop no	Latitude	Longitude	Altitude (m)	Locality type	Aspect	Slope	Marked plants	Pop size
1	28°31.746'	84°19.135'	2275	Low open	S	10°	101	450
2	28°33.024'	84°16.040'	2630	Low forest	NE	8°	159	>1000
3	28°31.573'	84°18.208'	2664	Low open	S	2°	130	>1000
4	28°31.790'	84°18.162'	2677	Low forest	SE	28°	89	200
5	28°33.859'	84°12.784'	2729	Low forest	S	8°	145	>1000
6	28°33.289'	84°14.065'	2695	Low open	S	3°	175	>1000
7	28°36.152'	84°10.296'	3177	High open	NE	6°	188	>1000
8	28°36.376'	84°09.745'	3214	High open	E	15°	152	>1000
9	28°36.505'	84°10.043'	3222	High open	SE	3°	135	500
10	28°37.201'	84°08.259'	3255	High forest	E	10°	149	1000
11	28°36.713'	84°09.379'	3223	High open	SE	3°	135	>1000
12	28°37.686'	84°07.196'	3356	High forest	NE	5°	68	70

The four combinations of altitude (high vs. low) and habitat openness (open vs. forest) are referred to as locality type. Marked plants are the plants that were followed in the permanent plots in the demographic study. Pop size is the estimated total number of *S*. *nubicola* plants at the locality.

### Demographic data collection

At each locality, we established one permanent plot of approximately 10 × 20 m, where we tagged all *S*. *nubicola* plants of all stages (usually 100–200 individuals). We recorded the number of vegetative and flowering stems and the length of the longest stem for each individual at the time of flowering in August for two years (2014–2015). Every year, we also recorded the number of new seedlings and followed their growth and survival in the permanent plots. At the time of fruiting in October 2014, the seed production per flowering stem was estimated for 15–30 flowering stems that were evenly distributed among 5–7 randomly selected flowering plants at each studied locality. Only black or dark-brown hard seeds were counted. Moreover, 100 seeds were sown in two 0.5 × 0.5 m plots of at each locality in October 2014 to estimate seedling establishment. Control plots without seed addition were used to check for potential contamination from local seed sources. Seedling establishment per plot was calculated as the number of established seedlings in the sowing plot minus the number of established seedlings in the adjacent control plot, and this difference was divided by the number of sown seeds.

To estimate the ability of the species to survive in the seed bank, two nylon bags, each containing 50 seeds, were buried at each locality after seed maturation in October 2014. The bags were excavated after one year in August 2015. All the seeds had decayed by this time; therefore, we thus assumed that *S*. *nubicola* does not form a permanent seed bank. All new plants found at the localities were seedlings. We thus assumed that the species can reproduce only generatively and not clonally. Seedlings were defined as plants with only one thin vegetative stem that was up to 5 cm high and germinated during the year in which they were recorded. In one year, the seedling develops into a vegetative plant; in two years, it may develop into a flowering plant [38].

### Herbivore damage

In August 2014, we recorded herbivore damage for each tagged plant individual (100–200 plants in each of the 12 populations and 195–558 plant individuals for each of the four locality types in total) to assess the effect of herbivore damage on the performance of the individual plants. For each individual, herbivore damage of five different leaves (if available) along randomly chosen stems was recorded through visual inspection as percentages and averaged. The herbivore damage was recorded within 5% range categories (0–5%, 5–10%, 10–15%, etc.), and it was recorded by a single person to allow the estimates to be comparable. The most abundant damage type recorded for *S*. *nubicola* was caused by leaf chewers. As caterpillars from the Noctuidae family were most commonly found feeding on *S*. *nubicola*, we assumed that they were the main herbivores. Rarely, caterpillars were also recorded on the reproductive parts of the plants. However, due to the rare occurrence of this type of herbivory, this information was not analysed further.

### Modelling the effect of herbivore damage on population dynamics

First, we tested the effect of plant size, altitude, habitat openness and their interaction on herbivore damage with generalized linear mixed effect models (GLMMs) using the lme4 package in R [42]. Plant size was expressed as the logarithm of the product of the length of the longest stem and the number of stems per plant. This measure explained the variability in vital rates of *S*. *nubicola* well and was also previously used to describe plant size for related species with a similar growth form [25,43]. Altitude and habitat openness were used as categorical variables with values “high” vs. “low” and “open” vs. “forest”, respectively. Population was used as a random factor in the models.

The population dynamics of the species were modelled using a size-structured integral projection model (IPM) [43,44]. IPM is analogous to a matrix population projection model [22], in that it projects population structures over discrete time steps; however, the population is modelled as a distribution function rather than a population vector, and the continuous kernel replaces the discrete matrix [45]. The data on individual plants were used to describe the life cycle transitions between two yearly censuses. Plant survival, growth (size in *t+1*), flowering (yes/no) and number of flowering stems per flowering individual were modelled as functions of plant size at time *t*, altitude, openness and herbivore damage by GLMMs using package lme4 in R [42]. Error distributions and link functions were specified to correspond to standard logistic regressions for survival and flowering, ordinary least squares regressions for growth, and Poisson regressions for the number of flowering stems as previously performed, e.g., by [45]. Herbivore damage was treated as a continuous variable. Altitude, openness and herbivore damage and their interactions in year *t* were used as explanatory variables for predicting the probability of flowering and the number of flowering stems in year *t* (representing reproduction potential to *t+1*) and the survival and growth from *t* to *t + 1*. For each vital rate, the population was included as a random factor. The regression models were simplified by omitting parameters with P > 0.5 to avoid overfitting. The interaction terms were tested first, and if kept, all lower-level terms were also retained in the models as suggested by [25]. Further model simplification was not attempted because it has been found to reduce the reliability of predictions in previous studies [46].

Seed production per flowering stem and seedling establishment were used as constants based on mean seed production and seedling establishment recorded in the field for each of the four locality types as described above. Differences in seed production per flowering stem and the numbers of established seedlings between altitudes and habitat openness types were tested with GLMM with locality as a random factor. Seed production per flowering stem was log transformed to achieve a normal distribution, and the probability of seedling establishment was tested using a logistic regression. Because of problems with the precise relocation of tags on seedlings, we did not have enough data on seedling survival and growth to vegetative plants for each locality type. We thus decided to average the data from all populations and use the averaged values in all four locality types. Seedlings were almost never damaged by herbivores, and although we were aware that there might be differences in seedling growth among populations, we assumed that seedling growth did not affect the results of our study with respect to the effect of herbivore damage on population dynamics. The number of new vegetative plants was calculated as the product of the predicted probability of flowering and number of flowering stems (predicted by GLMM as described above), mean seed production, mean seed germination and mean seedling establishment in each of the four locality types. We assumed that the size distribution of new vegetative plants established from seedlings was normal and estimated the mean and variance from the data. The permanent plots were placed within relatively homogenous stands of *S*. *nubicola*. The number of seeds leaving the plots was thus equal to that coming into the plots by dispersal from neighbouring plants.

We used the IPM to explore how the effects of altitude, openness and herbivore damage on vital rates translated to effects on population dynamics, using the different projection kernels resulting from differences in altitude, openness and herbivore damage. The parameters were then modified one by one to investigate their effects on population dynamics. Herbivore damage was modified by 1% in a range between 0% and 30%, approximately corresponding to the values observed in the localities during our previous study [38]. For each kernel, we calculated the asymptotic population growth rate and the elasticity of the growth rate to perturbations in vital rates. The uncertainty in our population growth rate estimates (95% confidence intervals) was calculated by bootstrapping the original data 1000 times, constructing a new IPM and deriving the population growth rates [47,48].

## Results

### Factors affecting leaf herbivore damage

**Fig 2:**
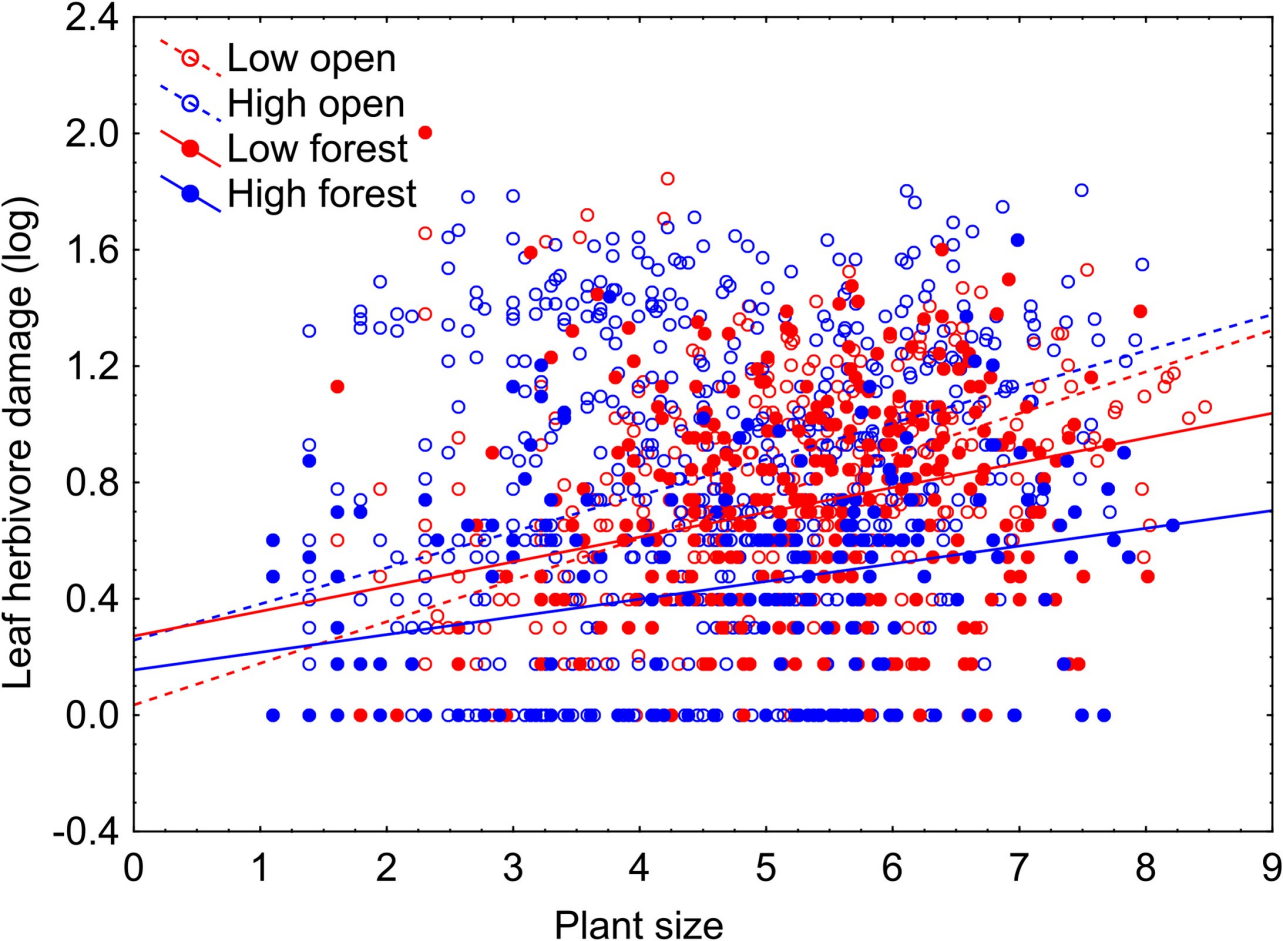
Relationship between plant size and leaf herbivore damage in four locality types of *Salvia nubicola*. Plant size was expressed as the logarithm of the product of length of the longest stem and the number of stems per plant. See Table 2 for details of the test results.

**Fig 3:**
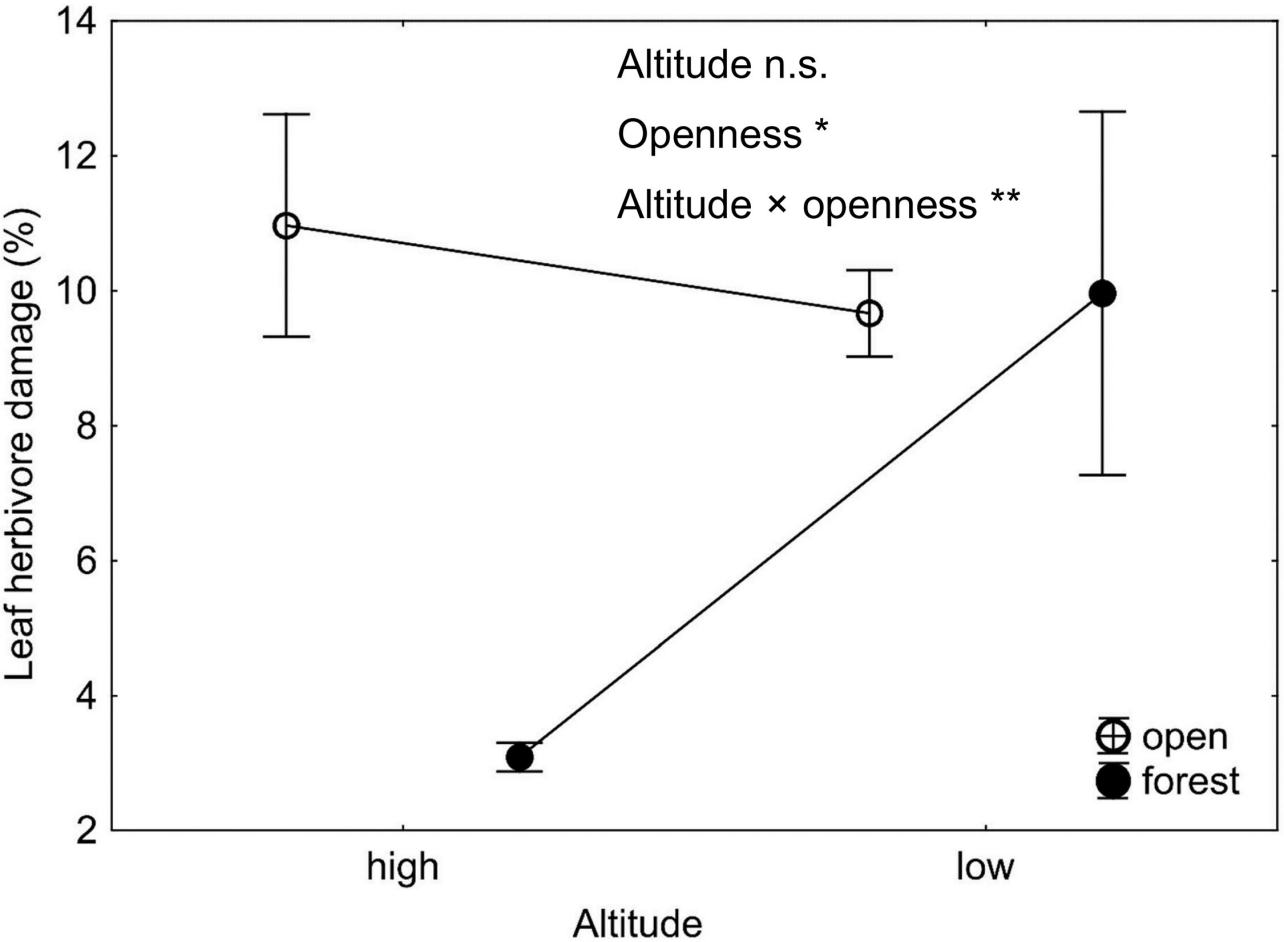
Differences in leaf herbivore damage among four locality types of *Salvia nubicola*. Altitude, openness and altitude × openness indicate the effect of altitude (low vs. high), habitat openness (open vs. forest) and their interaction. See Table 2 for details of the test results. * P < 0.05; ** P < 0.001; n.s. non-significant.

Leaf herbivore damage was affected by plant size, with larger plants being attacked more often (Table 2 and Fig 2). There was also higher herbivore damage in open compared to forest habitats. In forest populations, we found higher herbivore damage at low altitudes than at high altitudes. Contrary to our expectations, we did not find a similar pattern in open habitats where there were no differences between populations from different altitudes. This resulted in a significant interaction between altitude and habitat openness (Table 2 and Fig 3).

**Table 2:** Factors affecting herbivore damage recorded for individual plants of *Salvia nubicola*.

	Herbivore damage	
	F	P
Size	13.4	<0.001
Altitude	1.2	0.731
Openness	11.2	0.013
Size x altitude	1.0	0.240
Size x openness	2.7	0.075
Altitude x openness	9.3	0.002
Size x altitude x openness	1.2	0.289

Population code was used as a random effect in GLMMs (N = 1386).

### Factors affecting vital rates

**Fig 4:**
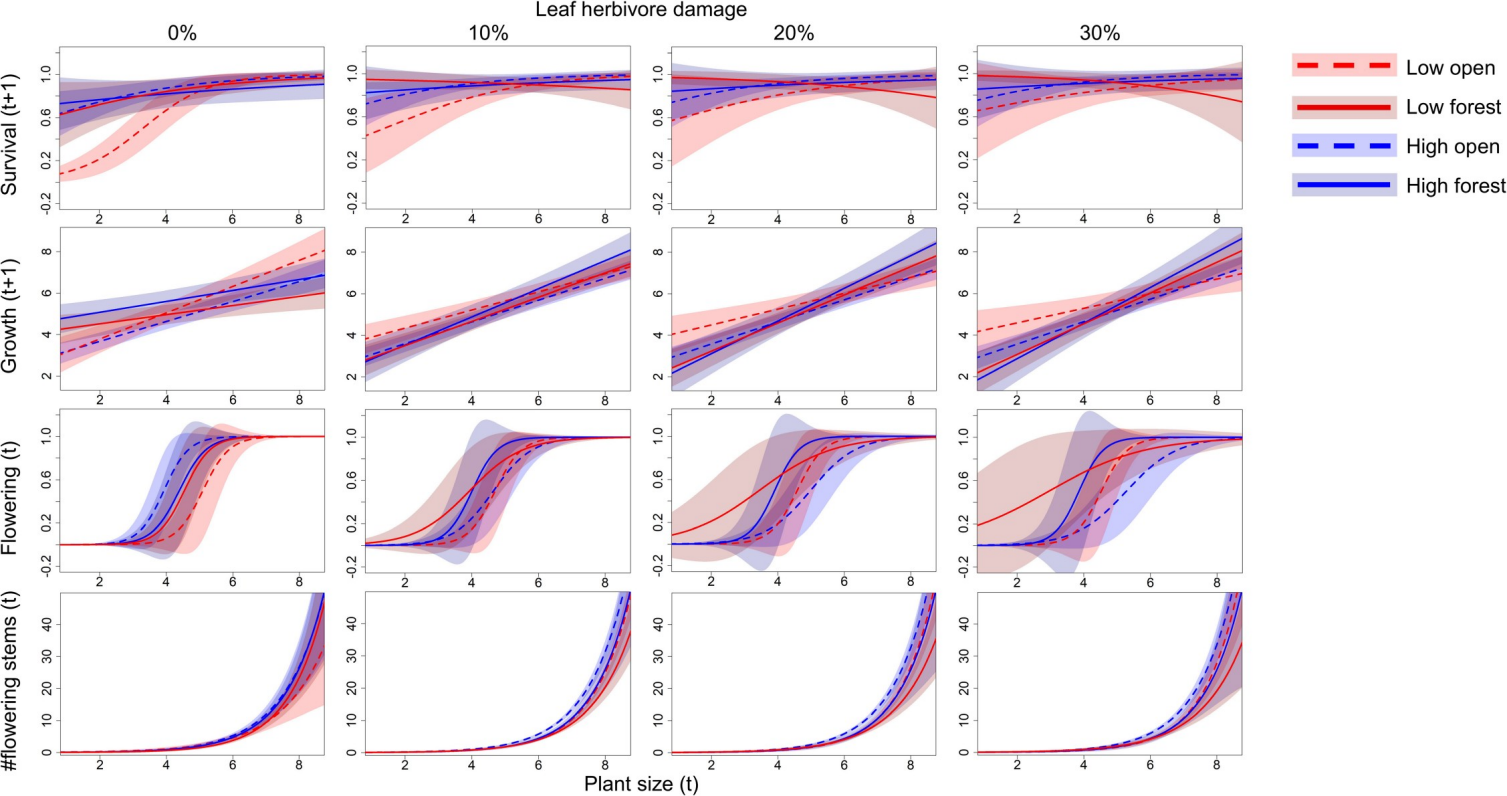
Relationship among plant size, herbivore damage and vital rates in four locality types of *Salvia nubicola*. Logistic regression was used for predictions of survival and flowering, ordinary least squares regression for growth, and Poisson regression for the number of flowering stems. Shading represents 95% confidence intervals of the predictions. Plant size was expressed as the logarithm of the product of the length of the longest stem and the number of stems per plant.

The survival of *S*. *nubicola* plants from time *t* to time *t+1* was affected by interactions between altitude and openness. There were also marginally significant interactions between altitude and herbivory and between size, altitude and herbivory (Table 3). All the interactions were caused primarily by populations in open habitats at low altitudes. While small compared to large plants had a lower probability of survival when no herbivore damage was present, increasing leaf herbivory caused even small plants to survive better in this locality type (Fig 4).

**Table 3:** Effects of plant size, altitude, habitat openness and herbivore damage on *Salvia nubicola* vital rates.

	Survival		Growth		Flowering		No. flowering stems	
	Chi	P	F	P	Chi	P	Chi	P
Size	1.30	0.254	**14.51**	**<0.001**	**17.64**	**<0.001**	**112.14**	**<0.001**
Altitude	0.36	0.547	0.71	0.399	0.05	0.830	0.57	0.450
Openness	0.59	0.441	**14.03**	**<0.001**	0.01	0.911	0.32	0.573
Herbivory	0.38	0.533	**13.86**	**<0.001**	0.00	0.967	0.06	0.811
Size × altitude	0.79	0.373	0.18	0.674	0.02	0.903	0.24	0.623
Size × openness	2.31	0.129	**5.81**	**0.016**	0.09	0.766	0.18	0.671
Size × herbivory	0.00	0.990	**10.79**	**0.001**	0.03	0.857	0.04	0.852
Altitude × openness	**5.81**	**0.016**	0.12	0.725	0.10	0.756	0.76	0.383
Altitude × herbivory	*3*.*76*	*0*.*053*	0.60	0.439	1.52	0.218	0.65	0.420
Openness × herbivory	0.08	0.767	**10.20**	**0.001**	0.23	0.634	0.01	0.934
Size × altitude × openness	2.61	0.106	1.57	0.210	0.06	0.815	0.73	0.394
Size × altitude × herbivory	*3*.*58*	*0*.*058*	0.11	0.745	1.49	0.222	0.57	0.450
Altitude × openness × herbivory	*		0.20	0.656	1.50	0.220	1.57	0.210
Size × openness × herbivory	*		**7.03**	**0.008**	1.01	0.316	0.01	0.946
Size × altitude × openness × herbivory	*		0.77	0.380	*2*.*84*	*0*.*092*	1.34	0.248

Tests were performed using GLMMs, and link functions were specified to correspond to logistic regressions for survival and flowering, ordinary least squares regressions for growth, and Poisson regressions for the number of flowering stems. Population was used as a random effect in all cases. Significant effects (P < 0.05) are in bold and marginally significant (P < 0.1) in italics. N = 1385 for survival and flowering, N = 1174 for growth and N = 777 for no. flowering stems.

* The regression model was simplified by omitting parameters with P > 0.5 to avoid overfitting. Interaction terms were tested first, and if kept, all lower-level terms were also retained in the models. Only results from the reduced model are presented.

Growth (plant size in time *t+1*) was strongly affected by plant size at time *t*, openness and herbivory. Plants in open compared to forest habitats and plants damaged by herbivores grew more when they were already large at time t. The interaction between plant size and habitat openness resulted from growth being more dependent on size in open compared to forest habitats. The interaction between plant size and herbivory showed that larger plants increased their growth after herbivore damage more than smaller plants did. This pattern was more obvious in forest habitats than in open habitats, which was indicated by a significant interaction between size, openness and herbivory (Table 3 and Fig 4).

The probability of flowering and the number of flowering stems per plant at time *t* strongly increased with plant size at time *t* (Table 3 and Fig 4). Marginally significant interactions among plant size, altitude, habitat openness and herbivory on the probability of flowering resulted from the higher flowering rate of small plants in forest habitats at low altitudes and lower flowering rate of small plants in open habitats at high altitudes in response to higher herbivore damage (Table 3 and Fig 4). Both seed production and seedling establishment were higher at low altitudes than at high altitudes (F = 5.0, P = 0.011, N = 436 and F = 7.9, P = 0.018, N = 24, respectively). While plants in open compared to forest habitats produced more seeds (F = 4.3, P = 0.029, N = 436), the pattern for seedling establishment was not consistently affected by habitat openness (F = 0.0, P = 0.978, N = 24) (S1 Table). The interaction between altitude and habitat openness was significant for neither seed production nor seedling establishment (P > 0.247). The probability of seedling growth to vegetative plants was 0.304 and was used as a constant for each of the four altitude openness combinations due to lack of data (see methods for details).

### Population performance in varying environments

**Fig 5:**
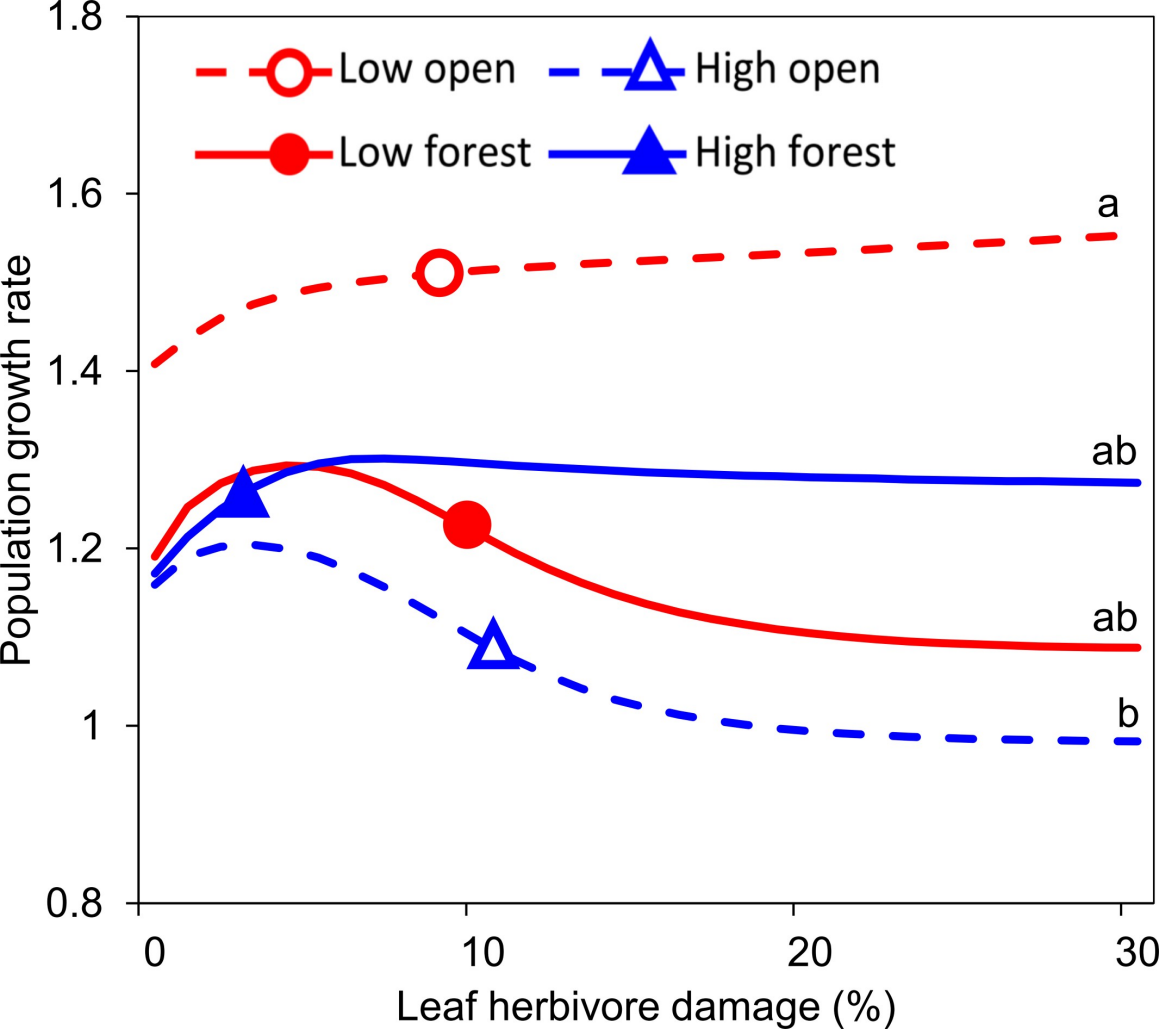
Effect of leaf herbivore damage on the population growth rate of *Salvia nubicola* populations. Open and filled circles and triangles represent values of mean insect herbivory that plants of all stages experienced at the respective locality types. Lines sharing the same letter are not significantly different from each other (P > 0.05).

Without herbivore damage, populations of *S*. *nubicola* grew (λ > 1) independent of altitude and habitat openness. The population growth rates in both types of high-altitude populations and in low altitude forest populations were very similar without herbivory (λ = 1.07–1.09). The open habitats at low altitudes had a higher population growth rate than the other environments (λ = 1.41). Herbivore damage, however, led to large differences in population growth rates (Fig 5).

In forest habitats, the changes in population growth due to herbivory were not significantly different between high and low altitudes (solid lines in Fig 5). However, populations in open habitats responded to increased herbivore damage differently. At low altitudes, the population growth rate consistently increased in response to increasing herbivore damage, and the populations from high altitudes responded to increasing herbivore damage negatively. This resulted in significant differences in population growth rates under high herbivore damage between populations from high and low altitudes in open habitats (dashed lines in Fig 5).

**Fig 6:**
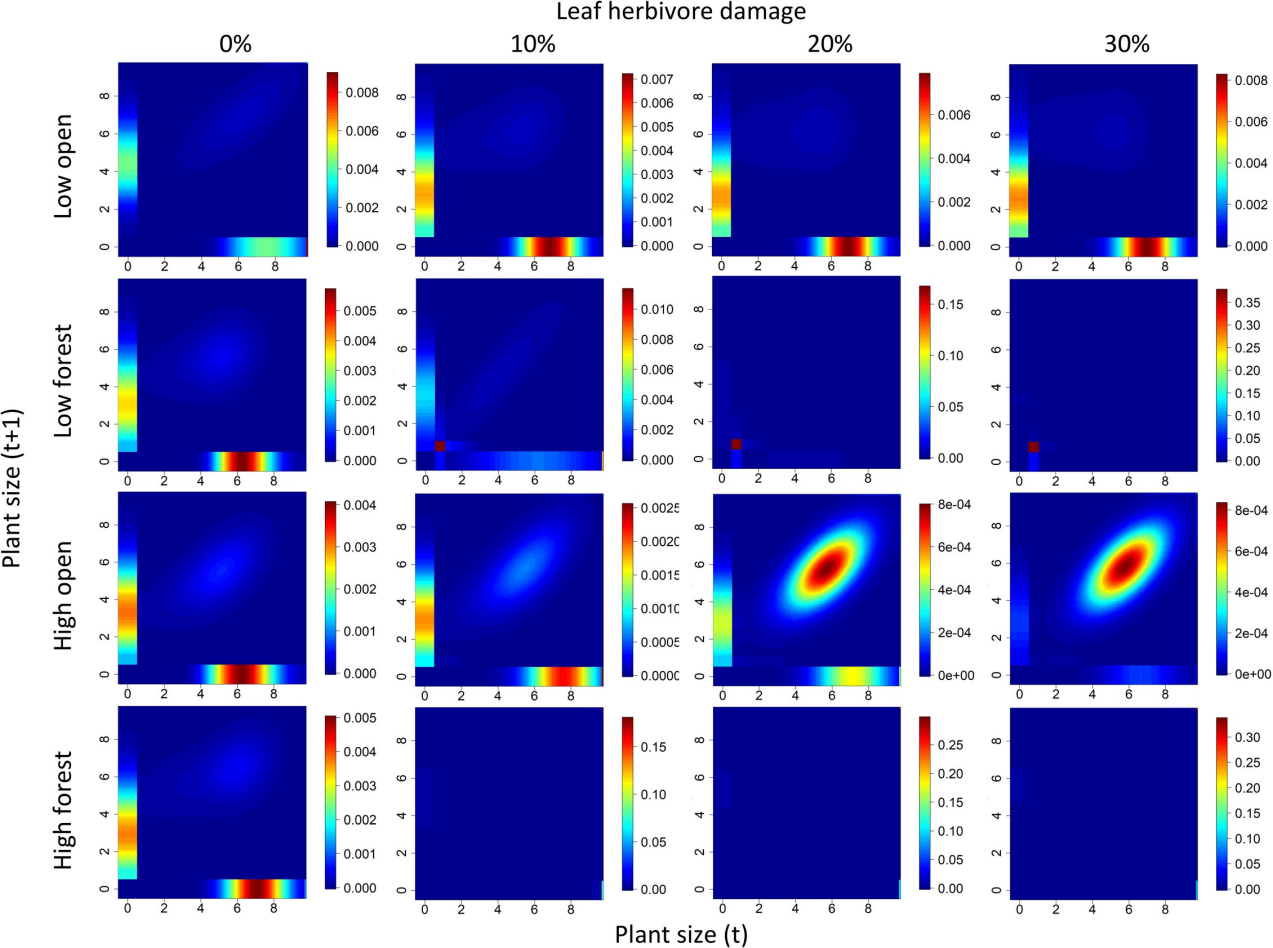
Elasticities in the four locality types of *Salvia nubicola* under increasing herbivore damage. Elasticity values indicate how different transitions between plants at time t and t+1 contribute to changes in the population growth rate. The lowest row within each panel shows the transitions between adult plants at time t and seedlings at time t+1 (seedling establishment). The first column within each panel shows the transitions between seedlings at time t and larger plants at time t+1 (seedling growth to larger plants). Plant size was expressed as the logarithm of the product of the length of the longest stem and the number of stems per plant. The dark blue colour indicates the lowest elasticity values, and dark red indicates the highest elasticity values.

Without herbivory, the populations at both altitudes and in both habitat openness types strongly relied on seedling establishment (horizontal bars along the x-axis on Fig 6) and their subsequent growth to larger plants (vertical bars in the first column on Fig 6), as indicated by the elasticity analyses. Differences in elasticity patterns appeared when herbivore damage was introduced to the populations (herbivore damage changed from 0 to 10%). While populations continued to rely on seedling establishment and seedling growth to larger plants at low altitudes in open habitats, the survival of small plants was the most important for population performance with increasing herbivore damage at low altitudes in forest habitats. At high altitudes, the survival of large plants (diagonals in Fig 6) was the most important for population performance in open habitats, while seedling establishment was the most important for population performance in forest habitats (Fig 6).

## Discussion

In line with our predictions, the population dynamics of *S*. *nubicola* depend on altitude, habitat openness, herbivory and their interactions. The populations perform quite well under the current levels of herbivory in both warmer and colder climates, represented by high and low altitudes. However, an increase in herbivore damage at high altitudes negatively affects the population growth rate. This effect is the case only in open habitats. Populations in forest habitats at high altitudes seem to deal with higher herbivore pressure much better than populations in open habitats.

### Herbivore damage

In agreement with other studies, we recorded higher herbivore damage for larger compared to smaller plants and for plants in open compared to forest habitats, as they are easier to find and colonize by the herbivores [49]. Large plants can also be more attractive to herbivores as they offer greater amounts and ranges of resources and niches [49]. This result is in line with the plant vigour hypothesis [50], which predicts that insect herbivores will preferentially choose larger and more vigorously growing plants. Warmer temperatures in open habitats can also lead to the greater activity of ectothermic animals, such as herbivorous insects [51].

Several studies found lower herbivore damage at higher altitudes [7,52,53]. Higher altitudes are characterized by harsher climates and/or limited food resources and habitat constraints [14] and are thus less suitable than lower altitudes for herbivores. In addition, the plants at high altitudes are usually less attractive and less palatable to herbivores because they are usually shorter (found also in our study, S1 Fig), tougher and produce various toxic secondary metabolites [8,38,54]. Higher herbivore damage at low altitudes was also found in the data from a wider range of populations of our study species [38]. Surprisingly, the pattern only held in the forest habitats in the current study. In open habitats, there were no differences in herbivore damage between populations from high and low altitudes (Fig 3). This result might be explained by the generally larger plants in forest habitats and especially the larger differences in plant size between low and high altitudes in forests compared to open habitats (S1 Fig). As discussed above, larger plants are more attractive to herbivores, and the larger differences in plant sizes between altitudes in forest habitats may thus explain the differences in herbivore damage. Leaves of plants growing in the shade are often more palatable and attractive to herbivores, leading to greater damage [55]. However, leaves exposed to full sunlight possess higher amounts of carbon-based secondary metabolites, such as phenolics and tannins, that are traditionally expected to deter the insects feeding on them [56,57]. All of these factors can cause larger differences in herbivore damage between low and high altitudes in forests compared to in open habitats.

### Population growth rates

Although insect herbivores are considered to have weak effects on plant population dynamics [58], multiple studies showed strong effects of insect herbivore damage on seed production and plant survival resulting in long-term decreases in population growth rates [31,59–61]. In our study, we recorded not only negative but also significantly positive effects of herbivory on plant performance. The effects were largely habitat specific in our study. Environmental conditions can affect plant growth directly through resource availability or indirectly by altering the behaviour or success of herbivores [37]. In our study, the plants were able to compensate for biomass loss along the whole altitudinal gradient in forest habitats, but there were large differences in the response to herbivore damage between populations at high and low altitudes in open habitats. In open habitats at low altitudes, plants survived and grew more in response to herbivory, and the population growth rate consequently increased in response to increased herbivore damage. This effect might be the result of overcompensation when plants benefit from being eaten, as the damage induces growth of the plants [62]. Although overcompensation is not very frequent, it has been observed in a wide variety of plant species (e.g., [62–64]). An alternative explanation for the positive relationship between the amount of herbivore damage and plant performance might be that herbivores prefer plants that are likely to perform better even if they are not consumed. In open compared to forest habitats, better-performing plants might be easier to recognize and colonize for herbivores. There may also be larger differences in plant performance in the open habitat due to higher habitat heterogeneity. Cornelissen et al. [65] showed a strong herbivore preference for more vigorous plants in a meta-analysis reviewing 71 published articles. Although we used plant size as a covariate in our analyses to control for differences in plant vigour, size does not necessarily have to be equivalent to plant vigour, and herbivores may be able to select more vigorous plants based on other cues. A study with experimentally manipulated herbivory would need to be conducted to verify this hypothesis. In open habitats at high altitudes, increased herbivory negatively affected flowering and consequently decreased the population growth rate. Strong effects of habitat on plant-insect interactions were also found in other studies [37,66,67]. Similar to our study, Hough-Goldstein and LaCoss [37] found that plants were better able to compensate for increased herbivory in favourable conditions, such as open places at low altitudes, than in unfavourable conditions. Salgado-Luarte et al. [66] found higher herbivory at the seedling stage of a temperate rainforest tree growing in a sunny compared to a shaded environment but also greater tolerance to herbivory in the sun than in the shade. In line with this, the plants in our study were able to best tolerate herbivory in open habitats at low altitudes, which are likely the most favourable conditions for our species due to the highest availability of light and highest temperatures in these otherwise rather extreme mountain environments [8,40]. Since plants from different altitudes and habitat types differ in their response to herbivore damage, it is probable that they not only differ in the extent of their response to herbivore damage but also in their defence strategies [68,69]. This result was confirmed in our previous study on the same species that demonstrated a trade-off among different types of defence strategies [8].

The population growth rate of the *S*. *nubicola* populations was mainly affected by seedling establishment and their subsequent growth to larger plants. This result is quite surprising since most similar perennial plants are characterized by a high elasticity of survival [70,71]. *S*. *nubicola* grows mostly in moist and nutrient-rich sites, which provide suitable conditions for the establishment of new plants. There is higher plant turnover indicated by the relatively high mortality of *S*. *nubicola* adult plants in the moist and nutrient-rich habitats than in the dry and less productive habitats studied in previous experiments [71,72].

Our prediction models were based on data from two years, i.e., one transition interval, when we had detailed data on the effects of herbivore damage on plant performance for all plants for which demographic data were available. There are multiple studies showing high variability in plant and insect population dynamics among years, e.g., [73–75]. However, we observed populations of *S*. *nubicola* for a year before this study began for the purpose of another study, and there were no large differences in the growth and survival of the plants among years. This is often the case in long-lived perennial plants, as they are usually not as sensitive to between-year variations in climate compared to other plants [25,71,76]. The population dynamics of such plants are usually affected by extreme climatic events, which are difficult to evaluate within studies over a few years [45]. A lack of temporal variability can also be solved to some extent by using multiple populations to provide information on spatial variability in different areas along an altitudinal gradient. Spatial variability was repeatedly shown to have stronger effects on population dynamics than temporal variability [77]. However, Jongejans et De Kroon [78] demonstrated that the relative magnitude of spatial and temporal variability was species dependent. The results of our study thus must be considered in this context.

## Conclusions

The studied populations of *S*. *nubicola* in both warmer and colder climates, represented by high and low altitudes, and in both habitats are expected to grow under the current levels of herbivory. However, an increase in herbivore damage at high altitudes might represent a serious threat to the populations in open, but not in forest, habitats. In our previous study [38], we recorded herbivore damage of approximately 30% for this type of *S*. *nubicola* population, which corresponded to the highest herbivore damage in our models. Populations from open habitats in high altitudes, especially in combination with extreme climatic and other stochastic events, might thus be at risk of becoming extinct. The species can, however, survive in forest habitats where the plants are better able to cope with higher herbivore pressure even at high altitudes.

In our study, we recorded unique data on the effects of herbivore damage at the individual plant level along a climatic gradient in field conditions. Although such experiments are very time consuming, we propose that such data are key for our ability to predict population responses to future climatic changes.

## Supporting information

S1 Table Seed production per flowering stem and seedling establishment of *Salvia nubicola* at different altitudes and habitat openness types. The means and their standard errors are shown. Different letters indicate significant differences among the four locality types (P<0.05).
(PDF)

S2 Table Primary data on the demography and herbivore damage to *Salvia nubicola*. (TXT)

S1 Fig Effect of altitude and habitat openness on the plant size of *Salvia nubicola*. Plant size was expressed as the logarithm of the product of the length of the longest stem and number of stems per plant. Boxes show means, standard errors and 1.96*standard errors.
(TIF)
