# Supplementary material for: Altitude, habitat type and herbivore damage interact in their effects on plant population dynamics
Source: PLoS One. 2018 Dec 17;13(12):e0209149. doi: 10.1371/journal.pone.0209149 (PMC6296709; doi:10.1371/journal.pone.0209149)
Supplement: S1 Table — The means and their standard errors are shown. Different letters indicate significant differences among the four locality types (P<0.05). (PDF) [file pone.0209149.s001.pdf]

S1 Table. Seed production per flowering stem and seedling establishment of *Salvia nubicola* in different altitudes and habitat openness types.

Altitude	Openness	Seed production	Seedling establishment
Low	Open	41.02 ± 3.70[a]	0.30 ± 0.08[a]
	Forest	33.40 ± 9.23[a]	0.23 ± 0.01[a]
High	Open	28.24 ± 4.90[a]	0.15 ± 0.03[b]
	Forest	14.75 ± 2.37[b]	0.19 ± 0.06[ab]

Means and their standard errors are shown. Different letters indicate significant differences between four locality types ($P<0.05$).
