# Supplementary figures and images for: Altitude, habitat type and herbivore damage interact in their effects on plant population dynamics
Source: PLoS One. 2018 Dec 17;13(12):e0209149. doi: 10.1371/journal.pone.0209149 (PMC6296709; doi:10.1371/journal.pone.0209149)

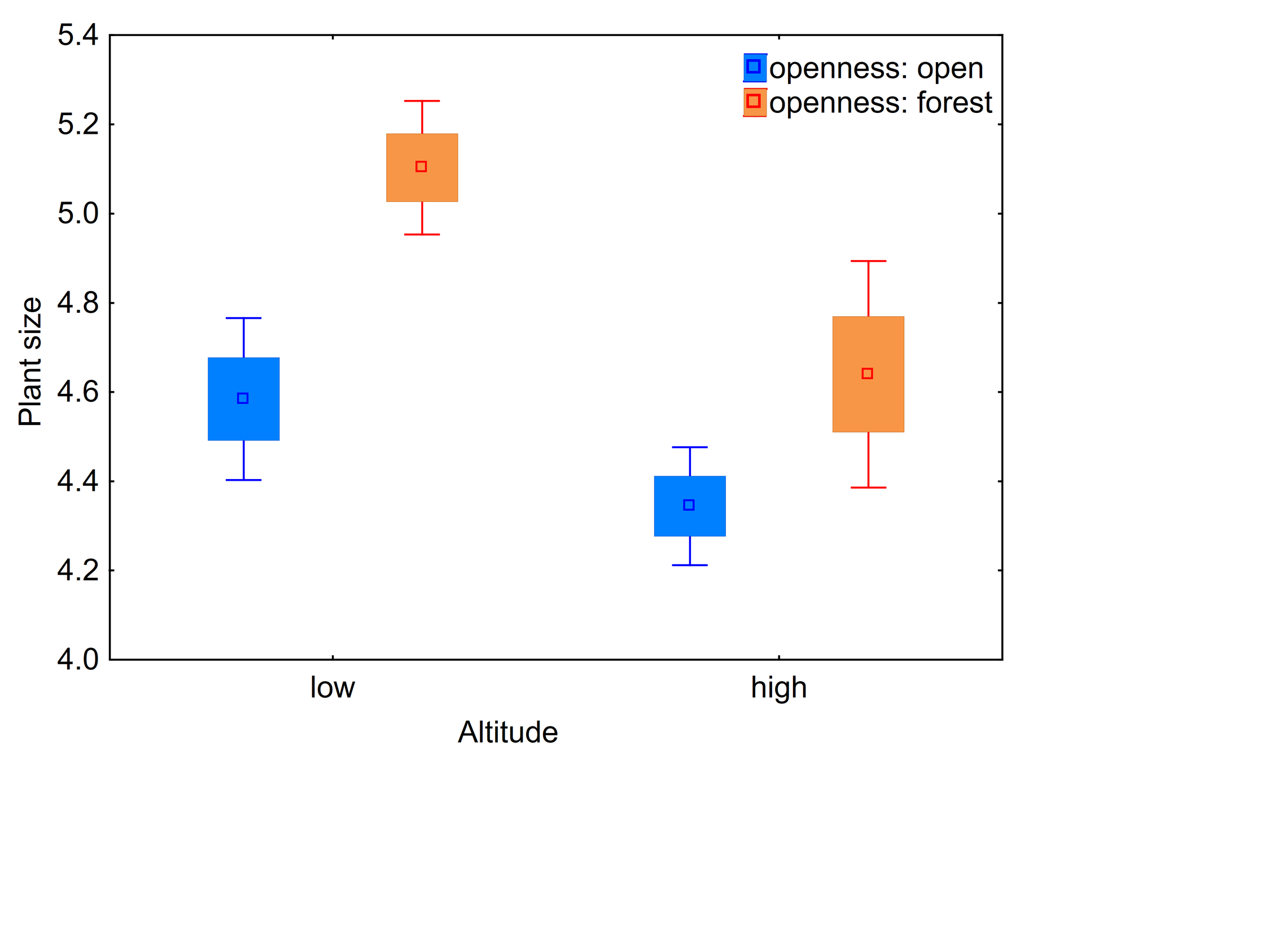

Supplement: S1 Fig — Plant size was expressed as the logarithm of the product of the length of the longest stem and number of stems per plant. Boxes show means, standard errors and 1.96*standard errors. (TIF) [file pone.0209149.s003.tif]
